# A simple null model for inferences from network enrichment analysis

**DOI:** 10.1371/journal.pone.0206864

**Published:** 2018-11-09

**Authors:** Gustavo S. Jeuken, Lukas Käll

**Affiliations:** Science for Life Laboratory, School of Engineering Sciences in Chemistry, Biotechnology and Health, KTH – Royal Institute of Technology, Box 1031, 17121 Solna, Sweden; Mayo Clinic Arizona, UNITED STATES

## Abstract

A prevailing technique to infer function from lists of identifications, from molecular biological high-throughput experiments, is over-representation analysis, where the identifications are compared to predefined sets of related genes often referred to as pathways. As at least some pathways are known to be incomplete in their annotation, algorithmic efforts have been made to complement them with information from functional association networks. While the terminology varies in the literature, we will here refer to such methods as Network Enrichment Analysis (NEA). Traditionally, the significance of inferences from NEA has been assigned using a null model constructed from randomizations of the network. Here we instead argue for a null model that more directly relates to the set of genes being studied, and have designed one dynamic programming algorithm that calculates the score distribution of NEA scores that makes it possible to assign unbiased mid *p* values to inferences. We also implemented a random sampling method, carrying out the same task. We demonstrate that our method obtains a superior statistical calibration as compared to the popular NEA inference engine, BinoX, while also providing statistics that are easier to interpret.

## Introduction

Over-Representation Analysis (ORA) is commonly used to infer function from sets of analytes such as genes, transcripts, proteins or metabolites [1–3]. The technique estimates which functional modules, such as complexes or pathways, are overrepresented among a set of identified analytes. One prominent application of the technique is expression analysis, where ORA is regularly used to assess alternation in pathway activity by examining significantly different concentrations of analytes between biological conditions, such as disease state or treatment group. Most ORA methods are assessing the overlap between the investigated set of analytes, the *query set*, and a functional module, the *pathway set*, using hypergeometric test or a Fisher’s exact test. However, variants such as Gene Set Enrichment Analysis (GSEA) [4] also includes information on expression levels of the analytes of the query set.

A limiting factor of ORA is the quality of the databases defining pathways. While http://pathguide.org list 708 databases of pathway definitions [5], some critique has been voiced concerning the completeness and rigour of current pathway databases. Hence, efforts have been directed to designing methods that extend the pathway definitions, by functional association networks, like STRING [6] and FunCoup [7]. Instead of directly examining the overlap between the query and pathway sets, one can evaluate the number of links in the functional association network that connect the query and pathway set [8–12]. We will refer to such methods as *Network Enrichment Analysis* (NEA), although the concept also is known as crosstalk analysis.

The significance of the inferences from NEA is assessed by null models that investigate the number of expected random links from the query gene set to the pathway. Previous efforts have settled for network randomization, where the network definitions are randomly constructed by methods with varying degrees of topological conservation in respect to the original network. Yet, the methods that are used for network randomization are often mentally untractable, as they include a big number of steps that are subject to complex constraints. As an example, in the link assignment plus second-order conservation method [10], links are assigned at random, but sequentially and at each step they must follow constraints that depend on previous link assignments. While this results in a network that is both randomized and contains some desired topological features, preserved from the original network, these nested constraints make it very hard to verify that the resulting network is representative.

In theory there is a space of all possible networks that could be made up from a certain number of nodes and node degree distribution, however, it is not clear that the link assignment and second-order conservation method samples evenly from that distribution. And, if the resulting networks are not representative, the rejection of the resulting null hypothesis is uninformative, defeating the purpose of the statistical test.

We argue that the network perturbation methods used in most flavours of NEA are missleading. Users of NEA do not differ from users of ORA, they want to test an association between a query set of genes and pathways. The typical user has used an experimental or computational method to derive a set of query genes and wants to know if it is a random set, or if it is enriched with respect to a pathway. In this question, the introduction of a network is not relevant. A network may help to increase the sensitivity of ORA methods, but for practical reasons it is better left out of the null hypothesis. Tests of significance in this type of analysis is naturally implemented as tests of the query set. That is, is the query set a randomly selected set of genes or is there an over-representation of well-connected genes in the query set?

Hence, here we employ a different null model, more directly aimed at modelling the lack of association between query and pathway gene sets, which we formulate as “there are not more links between the query and pathway gene sets than expected by chance”. Here, we present a dynamic programming algorithm, which we dubbed GeneSetDP, that calculates the exact score distribution of any query of a given size. We also implemented one random sampling algorithm for the same task, that we called GeneSetMC. Both algorithms circumvent the need of network pertubations for the calculations of significance. Combining the GeneSetDP and GeneSetMC with the scoring system of the popular method BinoX, we use simulations to demonstrate that both our algorithms produced unbiased statistics.

## Algorithms

In network-based gene set analysis one scores a query set of genes, $Q=\{g_{1}\ldots g_{Q}\}$, from a genome with genes, $G=\{g_{1}\ldots g_{G}\}$ (i.e. Q⊆G), and how they relate to a pathway, $P=\{p_{1}\ldots p_{P}\}$. In NEA the pathway maps to the genome through a network, *x*_*ij*_, where *x*_*ij*_ = 1 if *g*_*i*_ and *p*_*j*_ are connected, or *x*_*ij*_ = 0 otherwise. The pathways are scored by summing up all connections, i.e. the pathway score can be expressed as,

$$\begin{array}{c} s=\sum_{i=1}^{Q}\sum_{j=1}^{P}x_{ij}. \end{array}$$ (1)

We wanted to evaluate our score under the null hypothesis, *H*_0_: “There are not more links between the query and pathway gene sets than expected by chance”. This formulation translates to calculating the distribution of scores that would occur if the *Q* query genes are randomly selected from the genome, and see how frequently a score as extreme or more extreme than the current score would appear in that distribution. Hence, we wanted to determine a score distribution, *i.e*. the number of ways, *N*(*s*), we can pick *Q* genes and obtain a score, *s*, which we will give two methods for below. Given such a score distribution, we can express a mid *p* value [13, 14] for obtaining a score, *s*, as,

$$\begin{array}{c} p\left(s\right)=\frac{N\left(s\right)/2+\sum_{s^{\prime }=s+1}^{S}N\left(s^{\prime }\right)}{\sum_{s^{\prime }=0}^{S}N\left(s^{\prime }\right)}, \end{array}$$ (2)

where *S* is the maximal score, *s*, that *Q* genes can obtain from Eq (1).

We can reformulate Eq (1) by defining the number of links, $l_{i}=\sum_{j=1}^{P}x_{ij}$, which gives us a score,

$$\begin{array}{c} s=\sum_{i=1}^{Q}l_{i}. \end{array}$$ (3)

Such a number of links, *l*_*i*_, can be pre-computed for all genes in G. For later purposes we also define a mapping, *k*_*a*_ = ∑_{*i*:*l*_*i*_=*a*}_ 1, giving the number of genes having, *a*, links to the investigated pathway.

### Random sampling algorithm

We first implemented a random sampling algorithm to assess *N*(*s*). We randomly selected sets of *Q* genes from G. These sets scores were calculated with Eq (3). We counted the number of times, *F*_*B*_(*s*), a score of *s* was obtained when sampling *B* gene sets. We see that *F*_*B*_(*s*) will approximately have the same shape as *N*(*s*) when selecting a large *B*, and we can hence calculate *p* values by replacing *N* with *F*_*B*_ in Eq (2). We refer to this procedure as GeneSetMC.

### Computation of the score distribution

An alternative approach is to calculate the exact score distribution using dynamic programming. This can be done if we formulate *N*(*s*) as a recursion, by observing a partial sum function, *N*_*a*_(*s*, *c*), which expresses the number of ways to select a set of *c* query genes, each with ≤ *a* links to the pathway set and obtain a score of *s*. If we know the distribution of the number of ways to select genes with ≤ *a* − 1 genes, *N*_*a*−1_(*s*, *c*), for all *s* and *c*, *N*_*a*_(*s*, *c*) can be determined by investigating the ways to select genes with exactly *a* links to the investigated pathway. As there are $\left(\begin{array}{c} k_{a}\\ b \end{array}\right)$ ways to select *b* from *k*_*a*_ elements, there contribution to *N*_*a*_(*s*, *c*) from the selection of *b* genes with exactly *a* links are $\left(\begin{array}{c} k_{a}\\ b \end{array}\right)N_{a-1}\left(s-ab,c-b\right)$. When evaluating all possible values of *b* we see that

$$\begin{array}{c} N_{a}\left(s,c\right)=\sum_{b=0}^{k_{a}}\left(\begin{array}{c} k_{a}\\ b \end{array}\right)N_{a-1}\left(s-ab,c-b\right). \end{array}$$ (4)

We also see that *N*_*a*_(*s*, *c*) = 0 for all *s* < 0, *c* < 0 or *a* < 0, with the exception of *N*_−1_(0, 0) = 1.

The final score distribution is given by *N*(*s*) = *N*_*R*_(*s*, *Q*), where *R* = max_*i*_ *l*_*i*_, i.e the largest number of links to the pathway from any gene in the genome.

**Listing 1**. The central part of the dynamic programing algorithm for finding *N*(*s*). The function takes the vector of links per gene, *k*_*a*_, as well as the number of query genes, *Q* as an input. The function depends on two additional functions find_maxscore(k,Q), which calculates the maximal score a query of size *Q* can obtain, and comb(a,b), which calculates $\left(\begin{array}{c} a\\ b \end{array}\right)$.

**def** genesetdp (k, Q):

 max_s = find_maxscore (k, Q)

 N = np.zeros ((max_s+1,Q+1))

 N[0, 0] = 1

 **for** a **in range** (**len** (k)):

  **for** s **in range** (max_s, −1, −1):

   **for** c **in range** (Q, −1, −1):

    **for** b **in range** (k [a], 0, −1): *# Stop at b* = *1*

     **if** c−b>=0 **and** s−a*b>=0:

      N[s, c] += comb(k [a], b) * N[s−a*b, c−b]

 **return** N[:, q]

### Implementation

There is a memory efficient implementation. We first note that

$$\begin{array}{c} N_{a}\left(s,c\right)=\sum_{b=0}^{k_{a}}\left(\begin{array}{c} k_{a}\\ b \end{array}\right)N_{a-1}\left(s-ab,c-b\right)=\sum_{b=1}^{k_{a}}\left(\begin{array}{c} k_{a}\\ b \end{array}\right)N_{a-1}\left(s-ab,c-b\right)+N_{a-1}\left(s,c\right). \end{array}$$

This enables us to calculate *N* in-place, at least as long as we update the elements in *N*_*a*_ in a reverse order so that we do not alter elements from *N*_*a*−1_ still needed to update subsequent elements. That is, we do not have to copy the dynamic programming matrix in each iteration over *a*, instead, we can add $\sum_{b=1}^{k_{a}}\left(\begin{array}{c} k_{a}\\ b \end{array}\right)N_{a-1}\left(s-ab,c-b\right)$ to previous iterations *N*_*a*−1_(*s*, *c*), by nested updates over *s* ∈ {*S*, *S* − 1, …, 0} and *c* ∈ {*Q*, *Q* − 1, …, 0}. For details see Listing 1. We refer to this method as GeneSetDP.

## Methods

We downloaded network definitions and the BinoX software (on 2018-04-28) for comparisons from https://bitbucket.org/sonnhammergroup/binox. BinoX was run with the default parameters.

We also downloaded the NEA example given at the BinoX web site. The example files include a pathway definition file that groups 6819 genes into 289 human pathways and a network definition file that, after thresholding the links with a score of 0.7, gave 1244992 links between those genes.

For the purpose of demonstrating our argument, we selected a representative example in the “Glycolysis/Gluconeogenesis” pathway, as it contained a number of genes that coincided with the median number of genes of the pathways in the definition file.

## Results

We implemented a Python program that reads network and pathway definition files and scores a query sets against a pathway according to Eq (3), using GeneSetDP and GeneSetMC described in the Algorithm section, that enabled us to assign *p* values according to Eq (2). We downloaded pathway and network definitions from the BinoX’s website and used the same network threshold as BinoX default (0.7).

**Fig 1:**
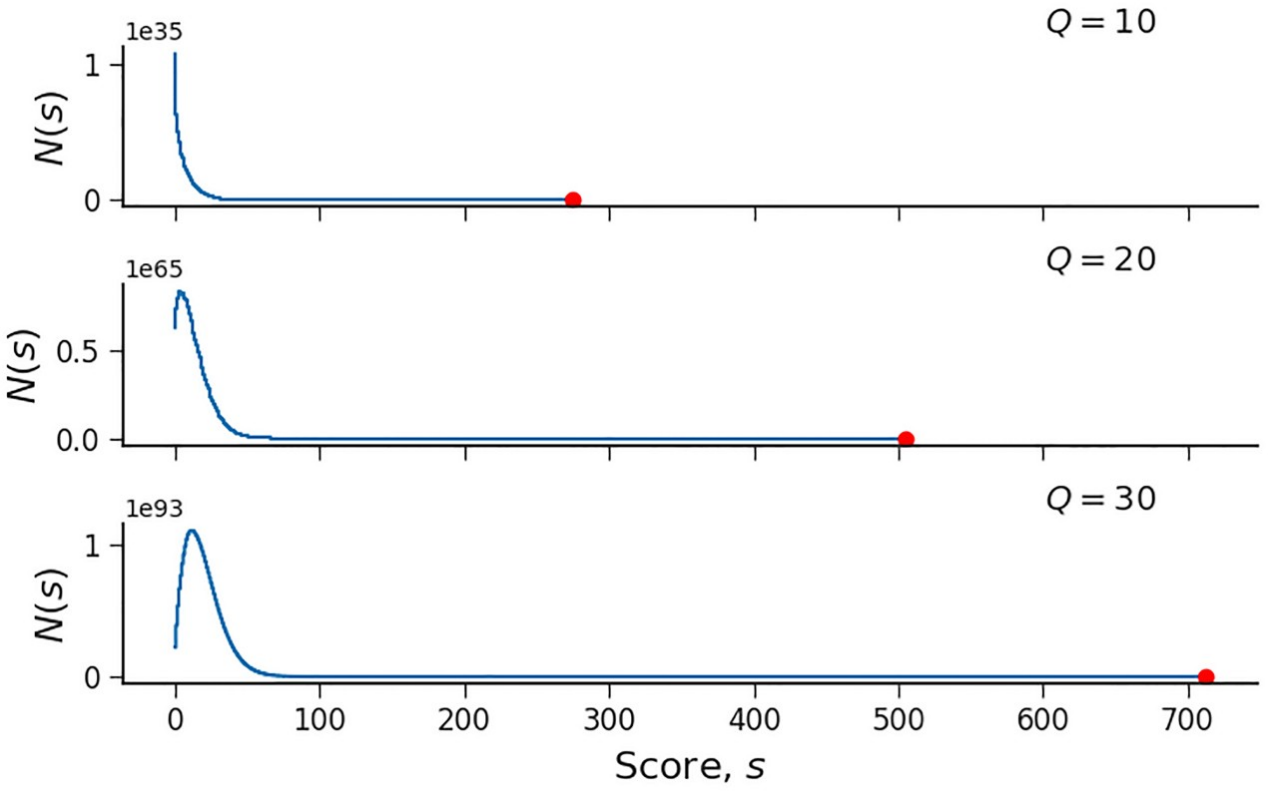
Score distribution of a query size of 10, 20 and 30 genes against the “Glycolysis/Gluconeogenesis” pathway. GeneSetDP enables us to calculate the full score distribution for a query of a given size, *Q*, i.e. how many ways one can reach a certain score when adding up the scores for *Q* genes. A red dot marks the maximum score, *S*, for each case.

To illustrate the *p* value calculation procedure, we plotted the score distributions *N*(*s*) for a query size of 10, 20 and 30 genes in Fig 1, using GeneSetDP.

### Test of calibration

**Fig 2:**
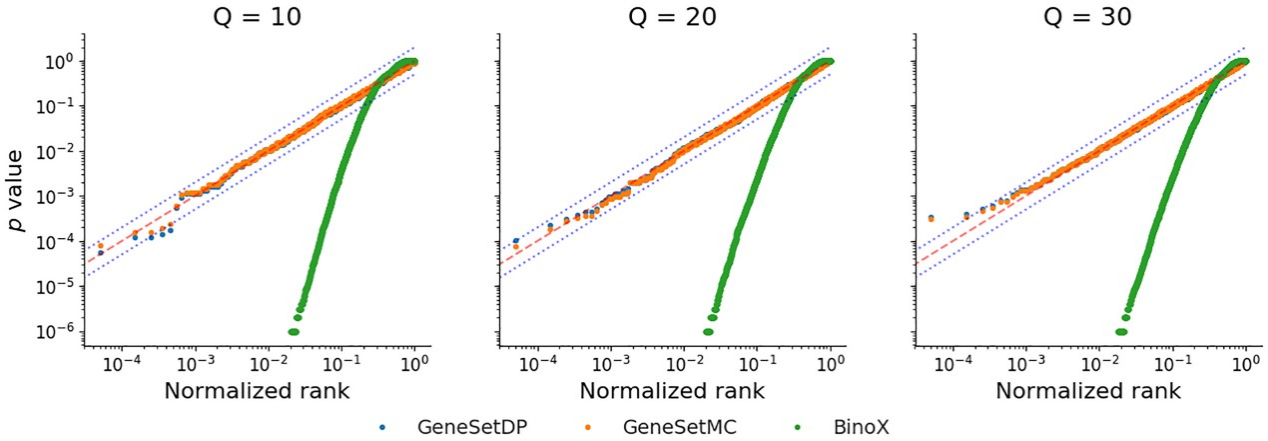
Calibration of GeneSetDP and GeneSetMC. We selected 10000 random sets of 10, 20 and 30 genes from the investigated genome definition and calculated their *p* values with GeneSetMC and GeneSetDP against the “Glycolysis/Gluconeogenesis” pathway. To test the *p* values uniformity we plotted them against their *normalized rank*, i.e. each *p* value’s (<rank>-0.5)/<total number of *p* values>. We also added the calibration curve of BinoX, evaluated on the same random sets. The dashed line shows *y* = *x* and the dotted lines *y* = 2*x* and *y* = 0.5*x*, for comparison.

In order to test the statistical calibration of our method, we calculated *p* values for 10000 random picks of gene sets of size *Q* from the investigated genome using both GeneSetDP and GeneSetMC with *B* = 100000. As our selection is random and following our null hypothesis, we expected the resulting *p* values to be uniformly distributed. To test this we plotted the *p* values against their quantile in Fig 2 for 10000 randomly assembled queries from the human genome. As a comparison, we also added a calibration curve, under our null hypothesis, for the popular NEA method BinoX [11]. Here, for all three methods, we are only testing the queries for enrichment, that is, we are only concerned with a higher number of links than expected by random, and this differs from the default BinoX setting which tests for both enrichment and depletion.

We note that the calibration of GeneSetDP is slightly conservative, i.e. the calculated *p* values are larger than expected. Meanwhile, BinoX appears strongly anti-conservative, that is, the *p* values are lower than expected, even when not taking into account significance by depletion.

**Fig 3:**
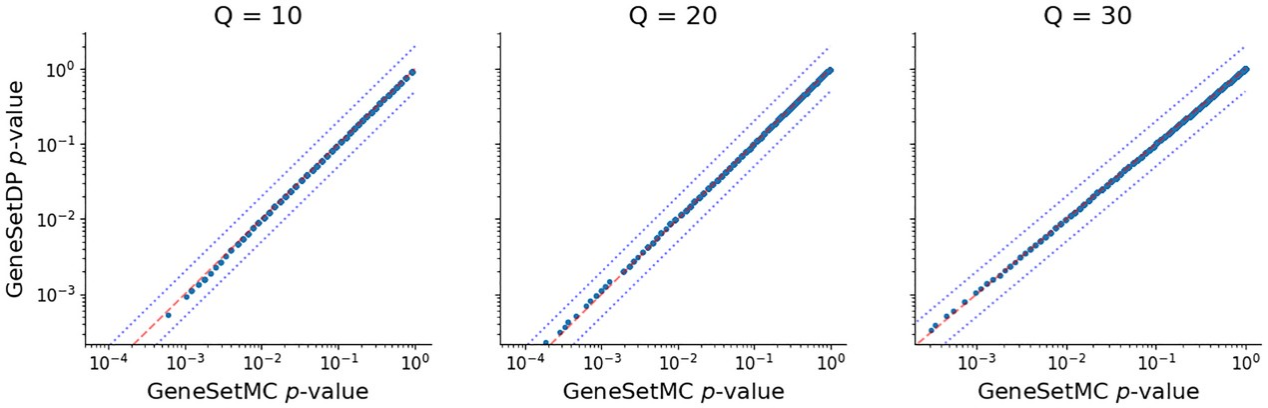
Correspondence between GeneSetDP’s and GeneSetMC’s *p* values. We selected 10000 random sets of 10, 20 and 30 genes from the investigated genome definition and calculated their *p* values with GeneSetMC and GeneSetDP against the “Glycolysis/Gluconeogenesis” pathway. We subsequently plotted the obtained *p* values against each other. Again, the dashed line shows *y* = *x* and the dotted lines *y* = 2*x* and *y* = 0.5*x*.

To test the consistency of the methods, we also investigated the correspondence between the *p* values reported by GeneSetDP and GeneSetMC. As can be seen in Fig 3, the results are consistent as expected.

## Discussion

Here we have implemented two methods, GeneSetDP and GeneSetMC, to calculate unbiased *p* values for inferences from NEA. Instead of testing our methods’ performance in terms of sensitivity, we here instead chose to test the method in terms of the methods statistical accuracy. We argue that more studies should make a point at demonstrating that their methods are well-calibrated, as it is a prerequisite for measuring performance, most importantly when the ground truth of biological effect is not known.

Both our implementations, GeneSetDP and GeneSetMC, are intended for measuring the enrichment of links between the query and the pathway set of genes. Most other NEA methods also report depletion of such links. One could easily modify our code to, instead of only measuring the higher scoring tail, measuring both tails of *N*(*s*). However, this might result in a drop in sensitivity, so we have so far not implemented such a mechanism.

We made an explicit definition of the null hypothesis we employed, which centers on the query, *H*_0_: “There are not more links between the query and pathway gene sets than expected by chance”. Previous implementations of NEA in the literature use network randomization methods to determine parameters for various types parametric distributions, by perturbations of the investigated network. In practice, such perturbations are very heuristic in their nature, and hence are difficult to use for calculating accurate statistics. Generally, it is easier to understand null models that relate to user actions.

Furthermore, network randomization methods focus on significance of the links in the network instead of the query set itself. In effect, NEA is an attempt to increase the sensitivity of ORA. The standard user of NEA would typically have identified a set of query genes which she wants to evaluate for over-representation in respect to a pathway database. This goal is the same as for users of ORA. Hence, the null model should be of the same nature for NEA and ORA. GeneSetDP and GeneSetMC are two algorithms that prove that one in NEA can test the uniqueness of the query set in respect to a pathway, just as done in ORA.
